# Hepatitis B Virus Covalently Closed Circular DNA Predicts Postoperative Liver Cancer Metastasis Independent of Virological Suppression

**DOI:** 10.3390/cancers13030538

**Published:** 2021-01-31

**Authors:** Chao-Wei Hsu, Yu-De Chu, Ming-Wei Lai, Chih-Lang Lin, Kung-Hao Liang, Yang-Hsiang Lin, Chau-Ting Yeh

**Affiliations:** 1 Liver Research Center, Chang Gung Memorial Hospital, Taoyuan 333, Taiwan; hsu2406@cgmh.org.tw (C.-W.H.); yudechu19871003@gmail.com (Y.-D.C.); a22141@adm.cgmh.org.tw (M.-W.L.); yhlin0621@cgmh.org.tw (Y.-H.L.); 2 Department of Hepatogastroenterology, Chang Gung Memorial Hospital, Taoyuan 333, Taiwan; 3 Division of Pediatric Gastroenterology, Department of Pediatrics, Chang Gung Memorial Hospital, Taoyuan 333, Taiwan; 4 Liver Research Unit, Chang Gung Memorial Hospital, Keelung 204, Taiwan; lion@cgmh.com.tw; 5 Medical Research Department, Taipei Veterans General Hospital, Taipei 112, Taiwan; khliang@vghtpe.gov.tw; 6 Molecular Medicine Research Center, Chang Gung University, Taoyuan 333, Taiwan

**Keywords:** hepatitis B virus, covalently closed circular DNA, peptide nucleic acid clamping, metastasis, hepatocellular carcinoma

## Abstract

**Simple Summary:**

The quantitative assessment of hepatitis B virus (HBV) covalently closed circular DNA (cccDNA) is essential to the development of next generation antiviral therapies against hepatitis B. Here, we developed a peptide nucleic acid (PNA)-clamping qPCR method to quantify cccDNA, which was comparable to the recently proposed exonuclease-based cccDNA assays. Using this method, we showed that cccDNA levels in the para-neoplastic liver tissues were independently correlated with overall survival, as well as extrahepatic metastasis in patients with or without virological suppression. These results suggest that in HBV-related hepatocellular carcinoma, patients under antiviral suppression might further benefit from new antivirals, which are designed to reduce cccDNA.

**Abstract:**

New antiviral therapies against hepatitis B virus (HBV) focus on the elimination of covalently closed circular DNA (cccDNA). However, traditional cccDNA-specific quantitative PCR (qPCR) has a narrow effective range, hindering a reliable comparison between the levels of biopsy-derived cccDNAs. Collaterally, the prognostic role of cccDNA in HBV-related hepatocellular carcinoma (HCC) cannot be clearly defined. Here, we developed a peptide nucleic acid (PNA)-clamping qPCR method to provide a wider range of specific cccDNA quantification (up to 5 logs of effective range). Extrachromosomal DNA was extracted from para-neoplastic tissues for cccDNA quantification. In total, 350 HBV-related HCC patients were included for an outcome analysis. Without differential pre-dilution, cccDNA levels in para-neoplastic liver tissues were determined, ranging from < 2 × 10^3^ to 123.0 × 10^6^ copies/gram. The multivariate linear regression analysis showed that cccDNA was independently correlated with the HBV e antigen (*p* < 0.001) and serum HBV-DNA levels (*p* = 0.012). The Cox proportional hazard model analysis showed that cccDNA independently predicted overall survival (*p* = 0.003) and extrahepatic metastasis-free survival (*p* = 0.001). In virologically suppressed HCC patients, cccDNA still effectively predicted intrahepatic recurrence-free (*p* = 0.003) and extrahepatic metastasis-free (*p* = 0.009) survivals. In conclusion, cccDNA independently predicted postoperative extrahepatic metastasis-free survival.

## 1. Introduction

Worldwide, hepatitis B virus (HBV) chronically infects an estimate of 240 million patients, resulting in an increased lifetime risk of cirrhosis, liver failure, and hepatocellular carcinoma [1,2]. In Taiwan, individuals who were born before 1986, the carrier rate is around 15% [1]. For those born after 1986, due to the success of the universal vaccination program, the prevalence rate decreases progressively to about 1% [3].

For patients with chronic HBV infection (CHB), two major types of approved therapeutics are available, interferon (IFN)-based and nucleos(t)ide analogs (NAs)-based treatments. These treatments have significantly improved the outcomes of CHB [4,5,6,7]. However, none of them can completely eradicate HBV, largely due to the persistence of biochemically stable HBV genomic templates, the covalent closed circular (ccc) DNA, in the hepatocytic nuclei. The prolonged treatment of NAs can prevent cccDNA replenishment, but cannot reduce the size of its existing pool. The IFN-based treatment can result in degradation of cccDNA, partly through the induction of APOBEC3s [8], but the process is quite slow and extensive side effects limited its long-term clinical use. Therefore, the current therapeutic goal in clinical practice is set as an optimal and sustained suppression of viral replication, so that hepatic inflammation can be ameliorated and the progression of fibrosis can be curbed. Accordingly, seroclearance or seroconversion of the hepatitis B e antigen (HBeAg), together with undetectable serum HBV-DNA, represent the “endpoints” of the treatment [9,10,11,12,13,14].

Inspired by a recent success of anti-hepatitis C virus treatment through direct acting antivirals, new goals of anti-HBV treatments are now designated as achieving seroclearance of HBV surface antigen (HBsAg), so that a “functional cure” can be reached. Several new anti-HBV regimens are under development, including entry inhibitors, cccDNA targeting agents, RNAi agents, secretion inhibitors, nucleocapsid assembly modulators, immune modulators, and therapeutic vaccine [15,16]. HBsAg serum concentrations have been correlated very well with cccDNA levels in HBeAg-positive but not HBeAg-negative patients [17,18]. However, the achievement of HBsAg clearance or seroconversion, representing the elimination of cccDNA, almost always occurs in patients with negative HBeAg (observed from negligible to 11% under approved antivirals) [19,20,21,22,23,24]. Therefore, the ideal marker for HBV elimination during new antiviral treatments remains to be the cccDNA level. Despite the fact that several surrogate serum markers have been proposed including HBV RNA level, HBV core-related antigen levels, etc., their correlation with cccDNA levels is moderate and does not surpass qHBsAg [15]. Finally, the direct measurement of intrahepatic cccDNA is unavoidable if a new drug is proven to completely eradicate HBV [25].

Current methods to quantify cccDNA remain clumsy. Since only a small piece of liver tissue can be obtained under liver biopsy, PCR-based quantitative methods (qPCR) are mandatory [26,27,28,29]. The “traditional” qPCR method for cccDNA relies on differential PCR efficiencies when the PCR is performed across the genomic gap of HBV relaxed circular DNA (rcDNA). When two primers are designed flanking the gap, the PCR is less efficient for rcDNA due to the presence of the genomic gap, but more efficient for cccDNA due to the absence of the gap [28,29]. However, due to the capability of nucleotide sequence jumping during the PCR, when the concentration of HBV-rcDNA is high, the rcDNA-related PCR product can still be produced. Therefore, it has been estimated that an effective differentiation between cccDNA and rcDNA can only be achieved between 1 to 10^3^ copies or less per the reaction mixture. When the total HBV-DNA concentration is high, extensive dilutions have to be made to allow the sample concentration to fall into the effective range. This maneuver not only caused an augmentation of assay variation, but also posted a major limitation when quantifying a smaller amount of cccDNA in a high level of total HBV-DNA. For example, if a sample contains 10^5^ copies of total HBV-DNA but 10^2^ copies of cccDNA, after dilution of the sample into the effective range, the cccDNA can no longer be detected. As a result, a fair comparison of cccDNA levels among the samples with various concentrations of intrahepatic HBV-DNA become almost impossible. To achieve a fair comparison, the qPCR method with a wider effective range to quantify cccDNA is urgently needed when developing next generation anti-HBV drugs.

In this study, we devised a new method using peptide nucleic acid (PNA)-clamping based qPCR to quantify cccDNA. PNA is a DNA mimic, where the negatively charged sugar-phosphate backbone is replaced with a neutral one containing repeated N-(2-aminoethyl) glycine units, which are linked by peptide bonds [30]. Due to the similar intra-molecular spacing, PNA can bind to DNA or RNA via Watson-Crick base paring. The PNA/DNA duplexes are very stable if no mismatch is present in the oligomers. The “PNA clamping” technique has been developed to identify single nucleotide polymorphism presented in low abundance [31,32,33]. Here, we used the PNA to “clamp” the single stranded region of rcDNA so that the PCR amplification can be inhibited, while cccDNA cannot be “clamped” (due to the existing complementary double strands), so that PCR can still be proceeded. With this method, an effective range of 0 to 10^5^ could be achieved. Subsequently, this method was applied to examine cccDNA levels in the para-neoplastic liver tissues from patients with hepatocellular carcinoma (HCC) and to correlate them with postoperative prognosis.

## 2. Materials and Methods

### 2.1. Patients and Samples

This study was conducted under the approval of Institutional Review Board, Chang Gung Memorial Hospital (IRB approval number: 201701780B0). In total, 350 para-neoplastic, non-cancerous liver tissues derived from HBV-related HCC patients (from September 2007 to 2014) were retrieved from Tissue Bank, Chang Gung Medical Center for tissue HBV-DNA and cccDNA quantification assays. Patients with a positive anti-hepatitis D antibody were not included in the present study. Clinical data were reviewed, including gender, age, anti-hepatitis C virus (anti-HCV) antibody, antiviral therapy used after operation, cirrhosis, alcoholism, ascites, HBeAg, serum HBV-DNA levels, alpha-fetoprotein (AFP), albumin, bilirubin, prothrombin time, creatinine, aspartate transaminase (AST), alanine transaminases (ALT), Child-Pugh functional class, maximum tumor size, tumor number, microvascular invasion, macrovascular invasion, Edmondson-Steiner histology grade, tumor capsule, Barcelona Clinic Liver Cancer (BCLC) tumor stage, immunohistochemistry (IHC) staining patterns of HBsAg, and IHC staining patterns of hepatitis B core antigen (HBcAg).

### 2.2. Quantification of cccDNA

**Figure 1:**
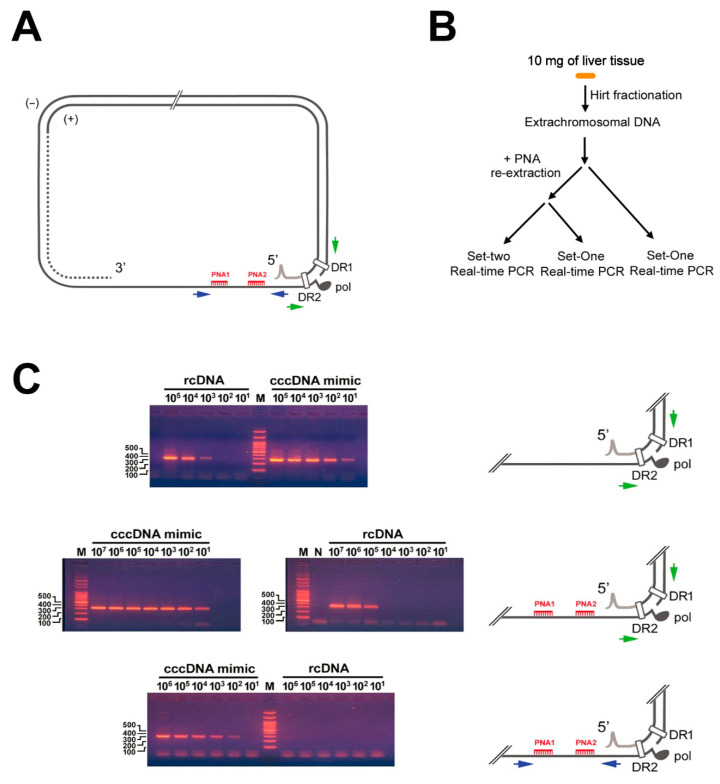
Schematic representation of peptide nucleic acid (PNA)-clamping based covalently closed circular DNA (cccDNA) assays. (**A**) Positions of all primers used. Red combs, two PNA primers, PNA1 and PNA2, used to “clamp” the single stranded region of minus-stranded hepatitis B virus (HBV)-DNA; green arrows, traditional cccDNA specific qPCR primers (Set-one primers); dark blue arrows, primers flanking the PNA-clamping primers (Set-two primers). Gray fragment, RNA fragment on the 5′-end of plus-stranded HBV-DNA; black solid oval, polymerase (pol) linked to the 5′-end of minus-stranded HBV-DNA. The areas of DR1 and DR2 were also marked. (**B**) Procedures for cccDNA quantification (see Methods). (**C**). Specific detection of cccDNA by the PNA clamping qPCR method. It was found that with no pre-PNA clamping, cccDNA specific primers (green), can only specifically detect cccDNA from 1 to 10^2^ copies (upper). When preclamping is performed, cccDNA can be specifically detected up to 10^4^ copies using cccDNA specific primers and 10^2^ to 10^5^ copies using Set-two primers (dark blue).

Two PNA oligomers were synthesized for clamping of the minus-strand HBV-DNA: PNA1, NH2-CTGCGGAACTCCTAGC (nt. 1271 to 1286) and PNA2, NH2-GCGCACCTCTCTTTACGCGG (nt. 1524 to 1543). Quantitative PCR (qPCR) was performed using two sets of primers. Set-one was the traditional cccDNA specific primers crossing the gap: CCCL, 5′-CCGTGTGCACTTCGCTTCA-3′ (nt. 1575 to 1593) and CCCR, 5′-GCACAGCTTGGAGGCTTGA-3′ (nt. 1882 to 1864). Set-two was primers flanking the PNA oligomers, PNAU, 5′-CTCTGCCGATCCATACT-3′ (nt. 1256 to 1272) and PNAD, 5′-CGGACCGGCAGATGAGAAGG-3′ (nt. 1577 to 1558). The positions of these oligomers and primers are shown in Figure 1A.

To quantify cccDNA, three sets of assays were performed (Figure 1B). For the first set, 5 μL of liver tissue HBV-DNA was subjected to qPCR using the traditional cccDNA specific primers. For the second and third sets, tissue HBV-DNA was first mixed with 10 pmole of PNA1 and PNA2 oligomers in 100 μL of double-distilled water. The mixture was heated to 90 °C for 1 min, 70 °C for 5 min, then placed at room temperature for 30 min. The PNA-clamped or unclamped DNA was precipitated in acidic alcohol with 20 mg of glycogen. The mixture was then washed once with 300 μL of 70% alcohol, vacuum-dried, and dissolved in 6 μL of double-distilled water to remove the excessive PNA. Real-time PCR was performed with the desired primers in a volume of 20 μL. The temperature/time in each cycle was 95 °C/30 s, 94 °C/5 s, 60 °C/20 s, and 72 °C/30 s. In each batch of assay, qPCR was performed in parallel to a set of serial diluted standards with known concentrations of serum samples (for rcDNA), pre-measured by the Roche TaqMan Assay. A plasmid, pCMV-HBV [34], containing one copy of greater-than-unit-length of HBV genome was used to mimic cccDNA. Serial dilutions were also made to obtain various concentrations of cccDNA mimic (Figure 1C). In this study, the BioRad CFX96 Real-Time RCR Detection System (Bio-Rad Laboratories, Hercules, CA, USA) was used. The concentrations were then calculated according to the cycle numbers of the standards and the samples.

When PNA-clamping was not performed, cccDNA specific primers can only perform effectively in the range of 1 to 10^2^ copies. On the other hand, PNA-clamping followed by qPCR using Set-one and Set-two primers revealed that the Set-one primers could specifically detect/quantify cccDNA in the range of 1 to 10^4^ copies (in the 20 μL PCR mixture), whereas Set-two primers could specifically detect/quantify cccDNA in the range of 10^2^ to 10^5^ copies. Using this tool, we were able to specifically quantify cccDNA from 1 to 10^5^ copies without differential pre-dilutions of the samples. After the calculation, the sensitivity of these assays to detect cccDNA was 2.0 × 10^3^ copies/gram of tissue, the upper effective quantitative limit was 2.0 × 10^8^ copies/gram. The intra-batch coefficient of variation was found to be <5%. The batch-to-batch coefficient of variation was determined to be <10%.

### 2.3. Assays for HBsAg, Anti-HCV, and Anti-Hepatitis D

HBsAg was measured by the radioimmunoassay (Ausria-II, HBsAg-RIA; Abbott Laboratories, North Chicago, IL, USA). The anti-HCV antibody was measured by a third-generation enzyme immunoassay (HCV EIA III; Abbott Laboratories). The anti-hepatitis D antibody was detected by the enzyme immunoassay (anti-HD; Formosa Biomedical Technology Corporation, Taipei, Taiwan).

### 2.4. Diagnosis Criteria for HCC and Postoperative Follow-Ups

The preoperative diagnosis of HCC was made by the following criteria. In cirrhotic patients, HCC could be diagnosed by one dynamic computed tomography or dynamic magnetic resonance imaging showing a nodule >2 cm with contrast uptake in the arterial phase and washout in venous or delayed phases, or two imaging techniques showing the aforementioned patterns for nodules 1–2 cm in diameter. A cytohistological confirmation was mandatory for patients who could not fulfill these criteria or for non-cirrhotic patients. Tumors were completely resected, with a safety-margin >1 cm. A postoperative follow-up was performed by ultrasonography, chest X-ray, AFP, and blood biochemistry every 1 to 3 months in the first year and every 3 to 6 months thereafter. Abnormal findings were verified by computed tomography or magnetic resonance imaging. An intrahepatic recurrence was established by the use of the aforementioned criteria. An extrahepatic recurrence was confirmed by biopsy, aspiration cytology, computed tomography, or magnetic resonance imaging, with the choice of study dependent on the location of the lesions, as well as the condition of the patient.

### 2.5. Western Blot Analysis for Twist1 and Slug

The Western blot analysis was performed as previously described [35]. The rabbit polyclonal antibodies against Twist1 (ab49254, Abcam, Cambridge, MA, USA) and Slug (PA5-20290, Thermo Fisher Scientific, Waltham, MA, USA) were used in 1:1000 and 1:500 dilution, respectively. The mouse monoclonal antibody against GAPDH (MAB374, Merck Millipore, Burlington, MA, USA) was utilized in 20000-folds dilution. Quantification of the expression levels was achieved by the use of the Image J software to measure the densitometry followed by normalization using the GAPDH level.

### 2.6. Statistical Analysis

Parametric data in normal distribution were presented as the mean ± standard deviation and compared by the student’s *t*-test. Numeric data not in normal distribution were presented as a median (range). Dichotomous data were presented as a number (%). The linear regression analysis was used to analyze cccDNA levels in relation to clinicopathological and virological parameters. The survival analysis was performed by Cox proportional hazard models and/or Kaplan-Meier analysis. For the latter, patients were grouped as having high and low values of a variable for the prognosis analysis. When needed, a cutoff was determined by the use of the minimal *p*-value approaches for clinical studies [36].

### 2.7. Tissue HBV-DNA Extraction and Quantitative Assay

The tissue HBV-DNA was extracted according to our previous publication [37]. Briefly, 10 mg of liver tissue was lysed in 1 mL of Hirt solution (0.6% SDS, 10 mM Tris-HCl (pH 7.5), and 10 mM EDTA). After cell lysis, 250 μL of 5 M NaCl was added to the solution and incubated overnight on ice. The mixture was centrifuged at 10,000 rpm for 40 min at 4 °C. The pellets (containing chromosomal DNA) were discarded. The supernatant was treated with proteinase K (150 μg/mL) and extracted twice with phenol, twice with phenol plus chloroform (1:1), and once with chloroform. DNA was precipitated with acid ethanol and dissolved in 100 μL of TE for further analysis. Subsequently, 50 μL of the extracted DNA was mixed with a 450 μL HBV-negative serum obtained from healthy controls. The HBV-DNA concentration was then assayed using the Roche TaqMan HBV Monitor (Roche Diagnostics, Basel, Switzerland). The lower limit of this test was 100 copies/mL (or 20 IU/mL). After the calculation, the lower limit for this assay using liver tissue was 2.0 × 10^4^ copies/g.

## 3. Results

### 3.1. Development of a Novel Method to Quantify cccDNA by the Use of PNA-Clamping

To improve the effective range of quantification for cccDNA, a PNA clamping-based, real-time qPCR was developed. In this assay, two PNA oligomers, PNA1 and 2, were synthesized to “clamp” the minus-strand HBV-DNA (Figure 1A). The qPCR was performed using two sets of primers. The Set-one primers (green arrows) were the traditional cccDNA-specific primers crossing the gap, while the other set (blue arrows, Set-two) were primers flanking the PNA oligomers, PNAU and PNAD (Figure 1A).

To assess the effective range and specificity of this newly developed method, the HBV rcDNA and cccDNA-mimic were diluted serially for this assay. As shown in Figure 1C upper panel, it was found that the effective range for the cccDNA specific qPCR (by Set-one) in the absence of clamping primers was only 1 to 10^2^ copies. However, following PNA1 and PNA2 clamping, the amplification of rcDNA was significantly inhibited. Combing the results using Set-one and Set-two primers after clamping, a wider effective range (up to 10^5^ copies) could be obtained (Figure 1C, middle and lower panels). Accordingly, a procedure was developed for the quantification of tissue cccDNA (Figure 1B).

### 3.2. Comparison between the PNA-Clamping Method with the Exonucleases-Based Methods to the cccDNA Assay

**Figure 2:**
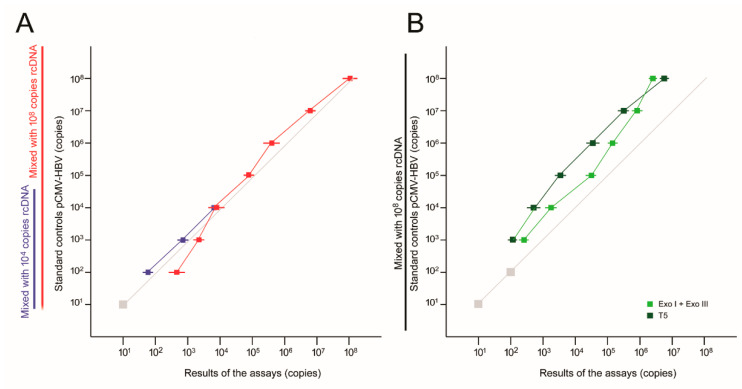
Comparison between the exonuclease based cccDNA assays and the PNA-clamping method. A total of 10^8^ copies of rcDNA were mixed with a series of cccDNA mimics (10^1^ to 10^8^ copies of pCMV-HBV, see Methods) for cccDNA quantification assays. (**A**) The PNA-clamping based assay was performed. Red lines and squares, mixed with 10^8^ copies rcDNA; blue lines and squares, mixed with 10^4^ rcDNA. (**B**) Exonuclease-based assays. Dark green lines and squares, T5 nuclease assay; light green lines and squares, exonuclease I and II assay. Gray line, the expected accurate results. Gray squares, undetectable concentrations. Three independent assays were performed for each concentration. Short horizontal lines, standard deviation.

Recently, exonuclease-based methods have been developed to quantify cccDNA. Exonucleases including exonuclease I and III, and T5 exonuclease were used to differentially digest away rcDNA before the quantification of cccDNA [38,39]. Here, we examined the performance of these two assays together with the PNA-clamping method by mixing high concentrations of rcDNA derived from the HBV-positive serum with cccDNA mimics (Figure 2). It was found that a relatively good linear correlation was maintained for the PNA-clamping based assay in the presence of 10^8^ copies rcDNA (Figure 2A). However, an overestimation was found when cccDNA was <10^4^ copies. This distortion was not observed if only 10^4^ copies rcDNA were mixed. For exonuclease-based assays, underestimation of the cccDNA concentrations was generally present (Figure 2B). In addition, when cccDNA < 10^3^ copies, it could not be detected.

### 3.3. Baseline Clinicopathological and Virological Data for HCC Patients Receiving Surgical Resection

**Figure 3:**
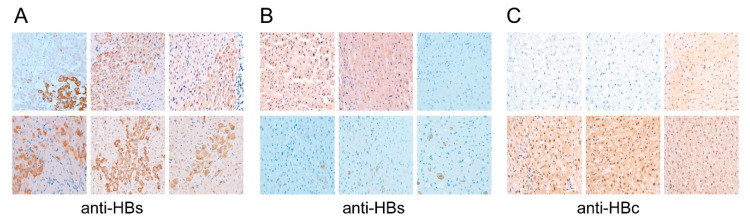
Distinct types of tissue distribution for HBsAg and HBcAg. (**A**,**B**) Tissue distribution patterns of HBsAg, (**A**) the clustered distribution pattern; ((**B**), upper left and middle) the diffuse distribution pattern; ((**B**), upper right) negative control; and ((**B**), lower panels) the scattered distribution pattern. (**C**) Tissue distribution patterns of HBcAg; ((**C**) upper left and middle) negative controls; ((**C**) upper right) predominantly cytoplasmic distribution; and ((**C**) lower panels) predominantly nuclear distribution. Antibodies used were given at the bottom: Anti-HBs: Antibodies against HBsAg; anti-HBc: Antibodies against HBcAg.

In order to apply this new approach for clinical use, a total of 350 liver tissue samples derived from para-neoplastic, non-cancerous liver tissues of HBV-related HCC patients receiving surgical operation were subjected for cccDNA and a total of liver tissue HBV-DNA quantification. Additionally, the tissue distribution patterns for HBV surface antigen (HBsAg) and HBV core antigen (HBcAg) were also analyzed following IHC staining. Distinct patterns of HBsAg and HBcAg distribution in tissues were found in these patients. For HBsAg, positive cells could be distributed in clusters (>5 positive cells adjacent to each other) (Figure 3A), spread diffusely throughout the section (Figure 3B, upper panels), or scattered widely (Figure 3B, lower panels). As for HBcAg, positive cells could be predominantly localized to the cytoplasm (nuclear HBcAg detected in <50% of positive cells; Figure 3C, upper right panel) or predominantly localized to the nuclei (nuclear HBcAg detected in > 50% of positive cells; Figure 3C, lower panels).

The basic clinicopathological and virological data were listed in Table 1. Of them, 36 (10.3%) were anti-HCV positive but HCV RNA negative (past infection). The antiviral therapy for HBV was given in 56 (16.0%) patients after the operation. In Taiwan, only the patients with liver cirrhosis and serum HBV-DNA level >10^4^ copies/mL were covered by health insurance for the antiviral therapy (implanted since June 2010). Patients who did not meet the criteria or who received an operation before implantation of the policy were not treated.

Following the quantification of tissue HBV-DNA and cccDNA, it was found that the median tissue HBV-DNA was 12.0 × 10^6^ copies/g (range, <2.0 × 10^4^ (lower limit of quantification) to 22455.5 × 10^6^ copies/g) and the median tissue cccDNA was < 2.0 × 10^3^ copies/g (range, <2.0 × 10^3^ (lower limit of quantification) to 123.0 × 10^6^ copies/g). Notably, 70/350 (20%) patients had tissue HBV-DNA < 2.0 × 10^4^ copies/g and 176/350 (50.3%) patients had tissue cccDNA <2.0 × 10^3^ copies/g. In the 174 patients who had tissue cccDNA > 2.0 × 10^3^ copies/g, the ratios of tissue HBV-DNA/cccDNA ranged from −1.42 to 3.10 log, which was within the effective range of this cccDNA assay. Of the 176 patients who had tissue cccDNA < 2.0 × 10^3^ copies/g, nine patients had tissue HBV-DNA > 10^9^ copies/g. The fact that these nine patients still had a result of cccDNA < 2.0 ×10^3^ copies/g, indicated that our new approach could indeed avoid a false positive of cccDNA.

### 3.4. Clinicopathological and Virological Factors Associated with cccDNA Amounts

To understand what parameters were associated with cccDNA levels in these patients, a linear regression analysis was conducted (Table 2). It was found that cirrhosis (*p* = 0.028), HBeAg (*p* < 0.001), pre-operative serum HBV-DNA (*p* = 0.005), BCLC tumor stage B (*p* = 0.028), positive tissue HBcAg (*p* = 0.003), and predominantly nuclear HBcAg (*p* = 0.001) were positively associated with cccDNA levels. Notably, the tissue HBV-DNA amount was only borderline significantly associated with cccDNA (*p* = 0.051), implying that the high levels of cccDNA might not proportionally be associated with high amounts of tissue HBV-DNA, but could lead to high concentrations of serum HBV-DNA. The multivariate linear regression analysis including the aforementioned factors showed that only HBeAg (*p* < 0.001) and pre-operative serum HBV-DNA (*p* = 0.012) remained to be significant.

### 3.5. Clinicopathological and Virological Factors Associated with Postoperative Prognosis

Subsequently, to understand which factors were associated with postoperative prognosis, including overall survival (OS), local recurrence free survival (LRFS), and extrahepatic metastasis free survival (EMFS), the Cox proportional hazard analysis was performed. In this study, 56/350 (16%) patients received antiviral therapy after the operation. As all these 56 patients became serum HBV-DNA < 100 copies/mL (lower detection limit for serum HBV-DNA concentrations) 1 to 3 months after the operation, one additional parameter, undetectable serum HBV-DNA 3 months post operation, was added for the analysis. Of the 350 patients included, 69 had HBV-DNA < 100 copies/mL 3 months after the operation.

For OS, the univariate Cox proportional hazard analysis showed that the postoperative undetectable HBV-DNA (*p* = 0.027), preoperative serum HBV-DNA (*p* < 0.001), albumin (*p* < 0.001), AST (*p* < 0.001), Child-Pugh functional class B (*p* = 0.032), tumor number (*p* = 0.038), microvascular invasion (*p* = 0.023), macrovascular invasion (*p* = 0.008), BCLC tumor stage B (*p* = 0.006), diffuse distribution of HBsAg (*p* = 0.016), tissue HBV-DNA (*p* = 0.021), and tissue cccDNA (*p* = 0.025) were significant predictors. The multivariate analysis showed that only albumin (*p* = 0.003), microvascular invasion (*p* = 0.050), diffuse distribution of HBsAg (*p* = 0.008), and cccDNA (*p* = 0.003), but not tissue or serum HBV-DNA, were independent predictors (Table 3). It was noteworthy that the diffuse distribution of HBsAg was associated with a better OS.

For LRFS, the univariate Cox proportional hazard analysis showed that the postoperative undetectable HBV-DNA (*p* = 0.027), ascites (*p* = 0.024), albumin (*p* < 0.001), Prothrombin time (*p* = 0.027), AST (*p* < 0.001), ALT (*p* = 0.029), microvascular invasion (*p* < 0.001), BCLC tumor stage B (*p* = 0.001), clustered distribution of HBsAg (*p* < 0.001), diffuse distribution of HBsAg (*p* = 0.018), positive tissue HBcAg (*p* = 0.002), and predominantly cytoplasmic HBcAg (*p* = 0.002) were significant predictors. The multivariate analysis showed that only microvascular invasion (*p* < 0.001), BCLC tumor stage B (*p* = 0.029), and clustered distribution of tissue HBsAg (*p* < 0.001) were independent predictors (Table 4). Notably, neither preoperative HBV-DNA, postoperative antiviral used, nor cccDNA were predictors for LRFS. In terms of HBV-DNA, only the postoperative undetectable HBV-DNA was important.

For EMFS, the univariate Cox proportional hazard analysis showed that the antiviral therapy after the operation (*p* = 0.023), postoperative undetectable HBV-DNA (*p* = 0.007), HBeAg (*p* = 0.001), AFP (*p* < 0.001), maximum tumor size (*p* = 0.011), tumor number (*p* = 0.011), microvascular invasion (*p* < 0.001), macrovascular invasion (*p* = 0.001), BCLC tumor stage B (*p* < 0.001), positive tissue HBcAg (*p* = 0.002), and tissue cccDNA amount (*p* = 0.005) were significant predictors. The multivariate analysis further showed that only AFP (*p* = 0.004), microvascular invasion (*p* < 0.001), macrovascular invasion (*p* = 0.042), and cccDNA amount (*p* = 0.001) were independent predictors (Table 5).

Taken together, these pieces of evidence suggested that cccDNA in liver tissues was an independent and important factor for predicting postoperative outcomes, especially the OS and EMFS.

### 3.6. Tissue Distribution Patterns of HBsAg and cccDNA Levels Predicted Postoperative Outcomes in Postoperative Serum HBV-DNA Negative Subgroup

**Figure 4:**
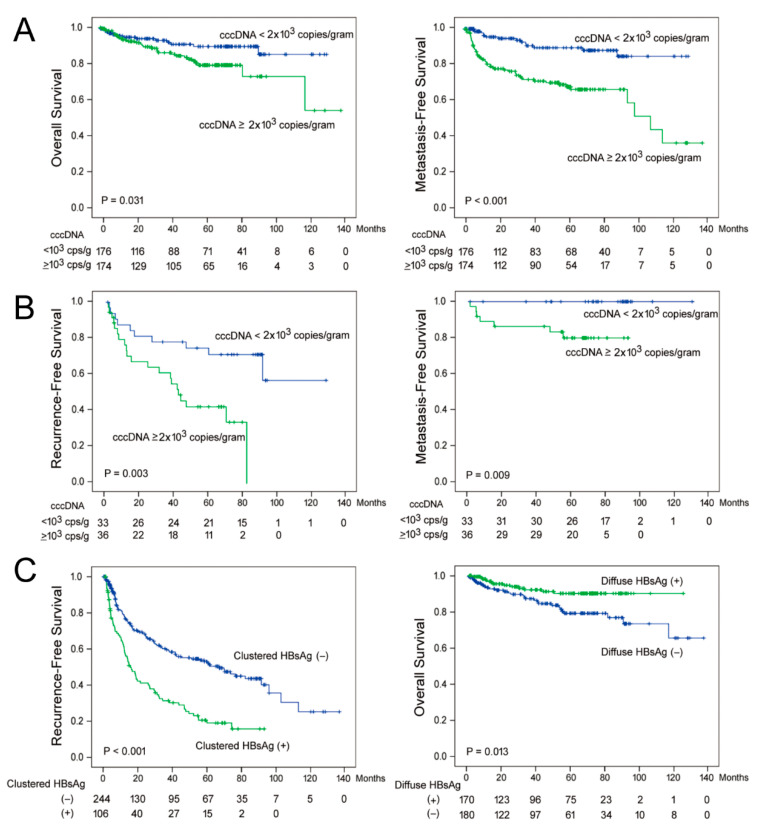
The association between postoperative outcomes and cccDNA levels. (**A**,**B**) Postoperative outcome comparisons between patients with low (blue curve) and high (green curve) cccDNA levels for all 350 patients (**A**) and 69 patients with undetectable serum HBV-DNA (**B**), respectively. (**C**) Postoperative outcome comparisons between patients with different tissue distribution patterns of HBsAg. The Kaplan-Meier analysis was conducted for all the comparisons.

As antiviral therapy could suppress serum HBV-DNA to an undetectable level, here, we asked whether the tissue cccDNA level was still associated with postoperative prognosis in patients that already achieved the undetectable serum HBV-DNA. The Kaplan-Meier analysis was conducted for all the patients (n = 350; Figure 4A) and for patients with undetectable postoperative HBV-DNA (n = 69; Figure 4B). It was found that when all the patients were included for analysis, cccDNA < 2.0 × 10^3^ copies/g was associated with a better OS (*p* = 0.031; Figure 4A, left panel) and EMFS (*p* < 0.001; Figure 4A, right panel), but not associated with LRFS (*p* = 0.103; not shown). This cutoff was selected since it was the lower limit of quantification and also the median value of cccDNA. When only patients with undetectable postoperative HBV-DNA were included, cccDNA < 2.0 × 10^3^ copies/g was associated with a better LRFS (*p* = 0.003; Figure 4B, left panel) and EMFS (*p* = 0.009; Figure 4B, right panel), but not associated with OS (*p* = 0.212; not shown). Of note, was that in the 33 patients who had undetectable postoperative serum HBV-DNA and tissue cccDNA < 2.0 × 10^3^ copies/g, none developed extrahepatic metastasis during the follow-ups (Figure 4B, right panel).

It was unexpected that tissue distribution patterns of HBsAg could predict postoperative LRFS by using the Cox proportional hazard analysis (Table 4). As a confirmation, the Kaplan-Meier analysis was also conducted. It was shown that a clustered distribution of HBsAg was associated with a shorter LRFS (*p* < 0.001; Figure 4C, left panel) and a diffuse distribution of HBsAg was associated with better LRFS (*p* = 0.013; Figure 4C, right panel) when all the patients were included. To examine whether the prediction remained effective in patients with negative postoperative HBV-DNA, we analyzed only this subgroup of patients. Intriguingly, a clustered distribution of HBsAg was also associated with a shorter LRFS in these patients (*p* < 0.001). However, a diffuse distribution of HBsAg was not associated with LRFS (*p* = 0.897).

### 3.7. The cccDNA Level Was Associated with Tissue Twist1 Expression Levels

**Figure 5:**
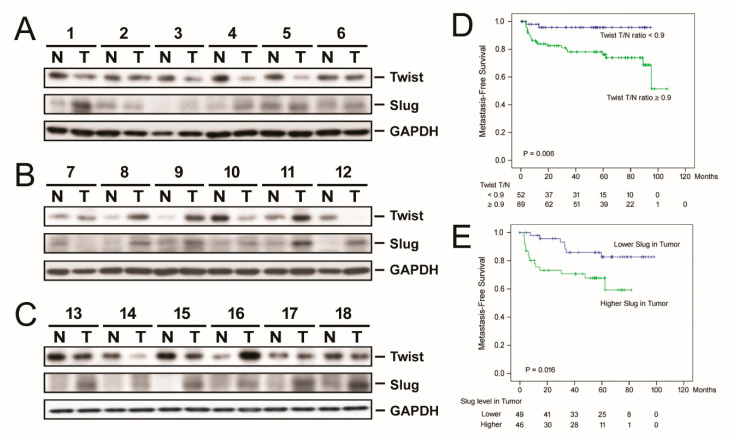
Association between the expression levels of epithelial-mesenchymal transition (EMT) markers, Twist1 and Slug, and postoperative outcomes in patients with HBV-related HCC. (**A**–**C**) Representative Western blots for an assessment of Twist1 and Slug expression levels in tissue samples derived from HCC patients. Densitometry was used for quantification. (**D**) Comparison of extrahepatic metastasis free survival (EMFS) between patients with high and low tumor/non-tumor (T/N) ratios of Twist1. Please find the western blot in Supplementary Files. (**E**) Comparison of EMFS between patients with high and low slug expression in cancerous tissues. The Kaplan-Meier analysis was performed. The cutoff of Twist T/N ratio (0.9) was determined by the minimal *p*-value approach [36].

Our present data indicated that the levels of tissue cccDNA were strongly associated with not only OS but also EMFS. As an attempt to search for the mechanism by which cccDNA could link to the extrahepatic metastasis of HCC, the association between cccDNA levels and the epithelial-mesenchymal transition (EMT) related proteins [40], Twist1 and Slug, was examined. Of the 350 patients, 141 and 95 had samples available for the Western blot analysis for Twist1 and Slug, respectively (Figure 5A–C). Intriguingly, the linear regression analysis showed that cccDNA levels in para-neoplastic, non-cancerous liver were positively associated with Twist1 levels in non-cancerous liver tissues (beta = 1.982 [95% CI, 1.341 to 2.622], *p* < 0.001) and in cancerous liver tissues (beta = 1.801 [95% CI, 1.062 to 2.540], *p* < 0.001). However, cccDNA levels were not associated with Slug levels (*p* = 0.921 in non-cancerous tissues and *p* = 0.976 in cancerous tissues). Moreover, it was found that a lower T/N ratio of Twist and a lower Slug level in the tumor were both associated with a better EMFS (*p* = 0.006 and 0.016, respectively; Figure 5D,E), albeit cccDNA levels were only associated with Twist1.

## 4. Discussion

A quantitative assessment of cccDNA in the para-neoplastic liver tissues to investigate its role in hepatocarcinogenesis is an important task, but it has rarely been thoroughly explored. Searching the literature, only a few reports were found [41,42,43]. Two of them used traditional cccDNA quantification methods, while the third used an invader assay but included only 25 patients. A major obstacle to complete such studies is the requirement of differential dilutions before performing a traditional cccDNA specific qPCR, leading to difficulties when attempting a fair comparison of cccDNA levels among the samples. In the present study, we took advantage of the PNA-clamping method, where a larger range (1 to 10^5^ copies) of effective cccDNA quantification could be achieved and therefore, no differential dilution was needed prior to the assays. Using this method, we discovered that cccDNA levels were associated with OS and EMFS. More importantly, in patients with a negative serum HBV-DNA (mostly due to the antiviral treatment), cccDNA levels were still associated with EMFS and LRFS. This finding was strongly argued for the need of new antiviral drugs to eliminate cccDNA following an optimal HBV-DNA suppression by NAs, as the postoperative prognosis might be further improved.

The mechanisms by which cccDNA could promote distant metastasis was unclear. In this study, we found that the cccDNA level was associated with the expression of Twist1, an important transcription factor associated with EMT, a process required for cancer cell metastasis [40]. A previous study has demonstrated that the HBV X (HBx) protein was associated with an increased Twist1 expression, which promoted the STAT control for EMT [44]. Since the increased cccDNA would lead to the increased generation of viral proteins, including HBx, it is likely that the cccDNA-associated augmentation of Twist1 expression is partly accomplished via HBx.

Another new finding that had never been reported was the association between HBsAg distribution patterns and HCC recurrence. The clustered distribution of HBsAg was associated with a shorter LRFS (Figure 3F). The association remained valid in serum HBV-DNA negative patients. We speculated that the presence of clustered distribution of HBsAg represented rigorous regeneration of multiple hepatocytic clones/micro-nodules in the cirrhotic liver, which contained differential sets of cellular factors, resulting in a better HBsAg expression in some cellular clones. Alternatively, it was highly likely that the HBsAg expressed in the clustered clones was originated from the integrated HBV-DNA, carrying oncogenic mutations. Presumably, liver experiencing rigorous regeneration and carrying cell clones/micro-nodules with aberrant HBsAg tends to have a higher risk of HCC recurrence after the operation, due to the accumulated somatic and/or viral protein gene mutations.

## 5. Conclusions

In summary, we developed a PNA-clamping based cccDNA assay, which did not require differential sample dilutions prior to the assay and was effective for cccDNA quantification over a wide range up to 5 logs. Using this method, we discovered that the cccDNA amounts were associated with postoperative distant metastasis and overall survival. Due to the fact that its association with distant metastasis was independent of the virological suppression advocated for further anti-cccDNA treatments in HCC patients, already achieving NA-based HBV suppression. 

## Figures and Tables

**Table 1:** Baseline data for 350 chronic hepatitis B patients with hepatocellular carcinoma.

Variable	Value
Clinical characteristics	
Sex, male/female, n (%)	282 (80.6%)/68 (19.4%)
Age, years, mean ± SD	53.5 ± 13.4
Anti-HCV positive ^a^, n (%)	36 (10.3%)
Antiviral therapy used after operation, n (%)	56 (16.0%)
Cirrhosis, n (%)	209 (59.7%)
Alcoholism, n (%)	108 (30.9%)
Ascites, n (%)	26 (7.4%)
Serology	
HBeAg, n (%)	18 (5.1%)
Serum HBV DNA, ×10^6^ copies/mL ^b^, median (range)	0.758 (0, 149.6)
AFP, ng/mL, median (range)	25.0 (0.9, 685353)
Albumin, g/L, mean ± SD	4.0 ± 0.6
Bilirubin, mg/dL, mean ± SD	1.1 ± 1.3
Prothrombin time, s, mean ± SD	12.2 ± 1.5
Creatinine, mg/dL, mean ± SD	1.1 ± 1.1
AST, U/L, mean ± SD	66.7 ± 87.0
ALT, U/L, mean ± SD	71.2 ± 94.7
Child-Pugh functional class	
A/B, n (%)	323 (92.3%)/27 (7.7%)
Tumor characteristics	
Maximum tumor size, cm, mean ± SD	6.2 ± 5.6
Tumor number ≤ 3, n (%)	330 (94.3%)
Microvascular invasion, n (%)	119 (34.0%)
Macrovascular invasion, n (%)	50 (14.3%)
Histology grade, mean ± SD	2.7 ± 0.7
Tumor capsule, n (%)	255 (72.9%)
BCLC tumor stage	
0-A/B, n (%)	153 (43.7%)/197 (56.3%)
Virological characteristics in para-neoplastic tissues	
Positive tissue HBsAg, n (%)	331 (94.6%)
Clustered distribution of HBsAg, n (%)	106 (30.3%) ^f^
Diffuse distribution of HBsAg, n (%)	170 (48.6%) ^f^
Scattered distribution of HBsAg, n (%)	109 (31.1%) ^f^
Positive tissue HBcAg, n (%)	219 (62.6%)
Predominantly cytoplasmic HBcAg ^c^, n (%)	126 (36.0%)
Predominantly nuclear HBcAg ^d^, n (%)	93 (26.6%)
Tissue HBV DNA, ×10^6^ copies/g, median (range)	12.0 (0, 22455.5) ^e^
Tissue cccDNA, ×10^6^ copies/g, median (range)	0.0 (0, 123.0) ^e^

^a^ HCV-RNA was negative in these patients. ^b^ Assessed <1 month before operation. ^c^ Nuclear HBcAg detected in <50% of positively stained cells. ^d^ Nuclear HBcAg detected in >50% of positively stained cells. ^e^ The lower limit of quantification for tissue HBV DNA was 2 × 10^4^ copies/g; the lower limit of quantification for tissue cccDNA was 2 × 10^3^ copies/g. ^f^ These patterns could co-exist in the same patients in different areas of the liver tissues.

**Table 2:** Linear regression analysis of tissue HBV cccDNA in relation to clinicopathological and virological parameters.

Variable	Beta (95% Confidence Interval)	*p*
Clinical characteristics		
Sex, male = 1	0.474 (−2.152, 3.101)	0.723
Age, years	0.058 (−0.020, 0.135)	0.144
Anti-HCV positive ^a^ = 1	−2.530 (−5.941, 0.881)	0.145
Cirrhosis, positive = 1	2.360 (0.256, 4.465)	**0.028**
Alcoholism, positive = 1	−1.217 (−3.463, 1.030)	0.288
Ascites, positive = 1	−0.464 (−4.427, 3.499)	0.818
Serology		
HBeAg, positive = 1	12.193 (7.666, 16.719)	**<0.001**
Serum HBV-DNA, ×10^6^ copies/mL ^b^	0.158 (0.049, 0.267)	**0.005**
AFP, ×100 ng/mL	0.000 (−0.003, 0.002)	0.690
Albumin, g/L	0.397 (−1.427, 2.221)	0.669
Bilirubin, mg/dL	−0.462 (−1.261, 0.336)	0.256
Prothrombin time, s	−0.426 (−1.139, 0.286)	0.240
Creatinine, mg/dL	−0.309 (−1.272, 0.654)	0.529
AST, U/L	−0.001 (−0.013, 0.011)	0.916
ALT, U/L	0.003 (−0.008, 0.014)	0.600
Child-Pugh functional class, class B = 1	−1.107 (−5.001, 2.787)	0.576
Tumor characteristics		
Maximum tumor size, cm	−0.114 (−0.299, 0.071)	0.227
Tumor number	−0.894 (−1.896, 0.107)	0.080
Microvascular invasion, positive = 1	−0.173 (−2.367, 2.021)	0.877
Macrovascular invasion, positive = 1	1.370 (−1.596, 4.337)	0.364
Histology grade	−0.837 (−2.372, 0.698)	0.284
Tumor capsule, positive = 1	0.003 (−2.334, 2.340)	0.998
BCLC tumor stage, stage B = 1	−2.337 (−4.417, −0.256)	**0.028**
Virological characteristics in para-neoplastic tissues		
Positive tissue HBsAg, positive = 1	2.328 (−1.882, 7.275)	0.247
Clustered distribution of HBsAg, positive = 1	1.046 (−1.213, 3.305)	0.363
Diffuse distribution of HBsAg, positive = 1	1.056 (−1.082, 3.072)	0.347
Scattered distribution of HBsAg, positive = 1	1.141 (−2.834, 1.653)	0.605
Positive tissue HBcAg, positive = 1	1.078 (1.072, 5.315)	**0.003**
Predominantly cytoplasmic HBcAg ^c^, positive = 1	−0.024 (−2.197, 2.150)	0.983
Predominantly nuclear HBcAg ^d^, positive = 1	3.887 (1.570, 6.204)	**0.001**
Tissue HBV DNA, ×10^6^ copies/g	0.001 (0.000, 0.001)	0.051
Multivariate linear regression analysis		
Cirrhosis, positive = 1	1.813 (−0.244, 3.870)	0.084
HBeAg, positive = 1	10.913 (6.260, 15.565)	**<0.001**
Serum HBV-DNA, ×10^6^ copies/mL ^b^	0.135 (0.029, 0.241)	**0.012**
BCLC tumor stage, stage B = 1	−1.984 (−4.008, 0.039)	0.055
Positive tissue HBcAg, positive = 1	1.330 (−0.990, 3.651)	0.260
Predominantly nuclear HBcAg ^d^, positive = 1	1.033 (−1.571, 3.637)	0.436

^a^ HCV-RNA was negative in these patients. ^b^ Assessed < 1 month before operation. ^c^ Nuclear HBcAg detected in <50% of positively stained cells. ^d^ Nuclear HBcAg detected in >50% of positively stained cells. Bold, *p* < 0.05.

**Table 3:** Cox proportional hazard analysis for clinicopathological and virological parameters in relation to overall survival.

	Univariate		Multivariate	
Variable	Hazard Ratio (95% CI)	*p*	Hazard Ratio (95% CI)	*p*
Clinical characteristics				
Sex, male = 1	1.145 (0.531, 2.472)	0.730		
Age, per year increase	1.004 (0.982, 1.027)	0.714		
Anti-HCV positive ^a^ = 1	0.567 (0.175, 1.835)	0.344		
Antiviral therapy used after operation, Yes = 1	0.451 (0.177, 1.153)	0.096		
Postoperative undetectable HBV DNA ^b^, Yes = 1	0.348 (0.136, 0.890)	**0.027**	0.453 (0.173, 1.185)	0.107
Cirrhosis, positive = 1	0.840 (0.459, 1.535)	0.570		
Alcoholism, positive = 1	1.391 (0.748, 2.587)	0.297		
Ascites, positive = 1	2.361 (0.994, 5.611)	0.052		
Serology				
HBeAg, positive = 1	1.469 (0.453, 4.763)	0.521		
Preoperative serum HBV DNA, ×10^6^ copies/mL	1.031 (1.016, 1.047)	**<0.001**	1.013 (0.981, 1.046)	0.433
AFP, per 100 ng/mL increase	1.000 (1.000, 1.001)	0.376		
Albumin, per g/L increase	0.345 (0.217, 0.549)	**<0.001**	0.392 (0.212, 0.725)	**0.003**
Bilirubin, per mg/dL increase	1.063 (0.899, 1.258)	0.476		
Prothrombin time, per s increase	1.143 (0.969, 1.348)	0.113		
Creatinine, per mg/dL increase	0.304 (0.069, 1.338)	0.115		
AST, per U/L increase	1.004 (1.002, 1.006)	**<0.001**	1.000 (0.996, 1.004)	0.859
ALT, per U/L increase	1.000 (0.997, 1.003)	0.999		
Child-Pugh functional class, class B = 1	2.576 (1.085, 6.116)	**0.032**	0.905 (0.301, 2.722)	0.859
Tumor characteristics				
Maximum tumor size, per cm increase	1.024 (0.998, 1.051)	0.066		
Tumor number, per number increase	1.307 (1.016, 1.683)	**0.038**	1.161 (0.874, 1.542)	0.303
Microvascular invasion, positive = 1	2.036 (1.101, 3.763)	**0.023**	1.987 (0.999, 3.954)	**0.050**
Macrovascular invasion, positive = 1	2.605 (1.279, 5.307)	**0.008**	1.371 (0.592, 3.174)	0.461
Histology grade, per grade increase	1.487 (0.961, 2.302)	0.075		
Tumor capsule, positive = 1	0.743 (0.392, 1.407)	0.361		
BCLC tumor stage, stage B = 1	2.488 (1.294, 4.785)	**0.006**	1.933 (0.900, 4.155)	0.091
Virological characteristics in non-tumor parts				
Positive tissue HBsAg, positive = 1	2.806 (0.384, 20.484)	0.309		
Clustered distribution of tissue HBsAg, positive = 1	1.336 (0.710, 2.515)	0.370		
Diffuse distribution of HBsAg, positive = 1	0.447 (0.232, 0.860)	**0.016**	0.385 (0.191, 0.777)	**0.008**
Scattered distribution of HBsAg, positive = 1	1.411 (0.754, 2.641)	0.282		
Positive tissue HBcAg, positive = 1	1.685 (0.858, 3.306)	0.129		
Predominant cytoplasmic HBcAg ^c^, positive = 1	1.069 (0.563, 2.030)	0.838		
Predominant nuclear HBcAg ^d^, positive = 1	1.578 (0.854, 2.918)	0.145		
Tissue HBV DNA, per 10^8^ copies/g	1.009 (1.001, 1.017)	**0.021**	1.000 (1.000, 1.000)	0.670
Tissue cccDNA, per 10^6^ copies/g	1.017 (1.002, 1.032)	**0.025**	1.029 (1.010, 1.049)	**0.003**

^a^ HCV-RNA was negative in these patients. ^b^ Assessed 3 months after the operation, regardless of the antiviral therapy used; serum HBV-DNA < 100 copies/mL. ^c^ Nuclear HBcAg detected in <50% of positively stained cells. ^d^ Nuclear HBcAg detected in >50% of positively stained cells. Bold, *p* < 0.05.

**Table 4:** Cox proportional hazard analysis for clinicopathological and virological parameters in relation to intrahepatic recurrence free survival.

	Univariate		Multivariate	
Variable	Hazard Ratio (95% CI)	*p*	Hazard Ratio (95% CI)	*p*
Clinical characteristics				
Sex, male = 1	1.142 (0.779, 1.674)	0.497		
Age, per year increase	1.002 (0.991, 1.013)	0.712		
Anti-HCV positive ^a^ = 1	0.962 (0.598, 1.550)	0.875		
Antiviral therapy used after operation, Yes = 1	0.687 (0.458, 1.031)	0.070		
Postoperative undetectable HBV DNA ^b^, Yes = 1	0.570 (0.386, 0.842)	**0.005**	0.690 (0.460, 1.034)	0.072
Cirrhosis, positive = 1	1.173 (0.866, 1.588)	0.302		
Alcoholism, positive = 1	1.194 (0.875, 1.629)	0.264		
Ascites, positive = 1	1.784 (1.081, 2.946)	**0.024**	1.436 (0.841, 2.453)	0.185
Serology				
HBeAg, positive = 1	1.573 (0.853, 2.900)	0.147		
Preoperative serum HBV-DNA, ×10^6^ copies/mL	1.012 (0.993, 1.030)	0.214		
AFP, per 100 ng/mL increase	1.000 (1.000, 1.001)	0.380		
Albumin, per g/L increase	0.599 (0.461, 0.779)	**<0.001**	0.763 (0.563, 1.034)	0.082
Bilirubin, per mg/dL increase	0.896 (0.742, 1.032)	0.255		
Prothrombin time, per s increase	1.100 (1.011, 1.198)	**0.027**	1.041 (0.944, 1.149)	0.419
Creatinine, per mg/dL increase	0.932 (0.781, 1.111)	0.430		
AST, per U/L increase	1.003 (1.002, 1.005)	**<0.001**	1.002 (1.000, 1.004)	0.116
ALT, per U/L increase	1.001 (1.000, 1.003)	**0.029**	1.000 (0.998, 1.002)	0.924
Child-Pugh functional class, class B = 1	0.913 (0.482, 1.728)	0.780		
Tumor characteristics				
Maximum tumor size, per cm increase	1.011 (0.995, 1.038)	0.176		
Tumor number, per number increase	1.100 (0.948, 1.275)	0.209		
Microvascular invasion, positive = 1	2.414 (1.780, 3.275)	**<0.001**	2.136 (1.543, 2.956)	**<0.001**
Macrovascular invasion, positive = 1	1.274 (0.827, 1.962)	0.271		
Histology grade, per grade increase	1.139 (0.919, 1.412)	0.233		
Tumor capsule, positive = 1	1.030 (0.739, 1.435)	0.862		
BCLC tumor stage, stage B = 1	1.655 (1.227, 2.231)	**0.001**	1.429 (1.036, 1.969)	**0.029**
Virological characteristics in non-tumor parts				
Positive tissue HBsAg, positive = 1	1.102 (0.562, 2.161)	0.777		
Clustered distribution of tissue HBsAg, positive = 1	2.309 (1.710, 3.118)	**<0.001**	2.093 (1.524, 2.874)	**<0.001**
Diffuse distribution of HBsAg, positive = 1	0.699 (0.519, 0.940)	**0.018**	0.749 (0.545, 1.030)	0.075
Scattered distribution of HBsAg, positive = 1	0.831 (0.596, 1.160)	0.277		
Positive tissue HBcAg, positive = 1	1.679 (1.218, 2.315)	**0.002**	1.372 (0.923, 2.038)	0.118
Predominantly cytoplasmic HBcAg ^c^, positive = 1	1.602 (1.185, 2.166)	**0.002**	1.338 (0.924, 1.938)	0.123
Predominantly nuclear HBcAg ^d^, positive = 1	1.083 (0.782, 1.498)	0.632		
Tissue HBV-DNA, per 10^8^ copies/g	0.997 (0.986, 1.008)	0.572		
Tissue cccDNA, per 10^6^ copies/g	1.008 (0.997, 1.019)	0.156		

^a^ HCV-RNA was negative in these patients. ^b^ Assessed 3 months after the operation, regardless of the antiviral therapy used; serum HBV-DNA < 100 copies/mL. ^c^ Nuclear HBcAg detected in <50% of positively stained cells. ^d^ Nuclear HBcAg detected in >50% of positively stained cells. Bold, *p* < 0.05.

**Table 5:** Cox proportional hazard analysis for clinicopathological and virological parameters in relation to distant metastasis free survival.

	Univariate		Multivariate	
Variable	Hazard Ratio (95% CI)	*p*	Hazard Ratio (95% CI)	*p*
Clinical characteristics				
Sex, male = 1	1.222 (0.639, 2.335)	0.544		
Age, per year increase	0.996 (0.978, 1.013)	0.621		
Anti-HCV positive ^a^ = 1	0.757 (0.327, 1.752)	0.515		
Antiviral therapy used after operation, Yes = 1	0.377 (0.162, 0.876)	**0.023**	0.857 (0.099, 7.415)	0.857
Postoperative undetectable HBV-DNA ^b^, Yes = 1	0.341 (0.155, 0.750)	**0.007**	0.427 (0.058, 3.127)	0.402
Cirrhosis, positive = 1	0.775 (0.479, 1.254)	0.299		
Alcoholism, positive = 1	1.097 (0.658, 1.828)	0.722		
Ascites, positive = 1	0.660 (0.207, 2.104)	0.482		
Serology				
HBeAg, positive = 1	3.281 (1.622, 6.638)	**0.001**	1.547 (0.679, 3.525)	0.299
Preoperative serum HBV-DNA, ×10^6^ copies/mL	0.998 (0.954, 1.044)	0.920		
AFP, per 100 ng/mL increase	1.001 (1.000, 1.001)	**<0.001**	1.000 (1.000, 1.001)	**0.004**
Albumin, per g/L increase	0.671 (0.433, 1.038)	0.073		
Bilirubin, per mg/dL increase	0.862 (0.593, 1.254)	0.438		
Prothrombin time, per s increase	1.108 (0.965, 1.273)	0.146		
Creatinine, per mg/dL increase	0.381 (0.125, 1.164)	0.090		
AST, per U/L increase	1.002 (1.000, 1.005)	0.065		
ALT, per U/L increase	0.998 (0.994, 1.002)	0.268		
Child-Pugh functional class, class B = 1	0.997 (0.363, 2.742)	0.996		
Tumor characteristics				
Maximum tumor size, per cm increase	1.026 (1.006, 1.047)	**0.011**	1.021 (0.952, 1.095)	0.561
Tumor number, per number increase	1.313 (1.064, 1.620)	**0.011**	1.161 (0.921, 1.465)	0.207
Microvascular invasion, positive = 1	3.943 (2.419, 6.426)	**<0.001**	2.767 (1.620, 4.727)	**<0.001**
Macrovascular invasion, positive = 1	2.701 (1.534, 4.758)	**0.001**	1.915 (1.023, 3.583)	**0.042**
Histology grade, per grade increase	1.409 (0.990, 2.007)	0.057		
Tumor capsule, positive = 1	0.794 (0.473, 1.331)	0.381		
BCLC tumor stage, stage B = 1	2.719 (1.595, 4.637)	**<0.001**	1.536 (0.724, 3.258)	0.263
Virological characteristics in non-tumor parts				
Positive tissue HBsAg, positive = 1	2.156 (0.527, 8.826)	0.285		
Clustered distribution of tissue HBsAg, positive = 1	1.251 (0.753, 2.079)	0.387		
Diffuse distribution of HBsAg, positive = 1	1.136 (0.701, 1.838)	0.605		
Scattered distribution of HBsAg, positive = 1	0.732 (0.416, 1.291)	0.281		
Positive tissue HBcAg, positive = 1	2.591 (1.433, 4.686)	**0.002**	1.867 (0.995, 3.502)	0.052
Predominantly cytoplasmic HBcAg ^c^, positive = 1	1.532 (0.934, 2.511)	0.091		
Predominantly nuclear HBcAg ^d^, positive = 1	1.587 (0.971, 2.597)	0.066		
Tissue HBV-DNA, per 10^8^ copies/g	0.996 (0.975, 1.018)	0.734		
Tissue cccDNA, per 10^6^ copies/g	1.017 (1.005, 1.029)	**0.005**	1.026 (1.011, 1.042)	**0.001**

^a^ HCV-RNA was negative in these patients. ^b^ Assessed 3 months after the operation, regardless of the antiviral therapy used; serum HBV-DNA < 100 copies/mL. ^c^ Nuclear HBcAg detected in <50% of positively stained cells. ^d^ Nuclear HBcAg detected in >50% of positively stained cells. Bold, *p* < 0.05.

## Data Availability

The data presented in this study are available on request from the corresponding author. The data are not publicly available due to local Institutional Review Board regulation.

## Supplementary Materials

The following are available online at https://www.mdpi.com/2072-6694/13/3/538/s1, Supplementary Files: The western blot of Figure 5.
