# Supplementary material for: Hepatitis B Virus Covalently Closed Circular DNA Predicts Postoperative Liver Cancer Metastasis Independent of Virological Suppression
Source: Cancers (Basel). 2021 Jan 31;13(3):538. doi: 10.3390/cancers13030538 (PMC7867012; doi:10.3390/cancers13030538)

Supplement western blots (original blots)

Because of the limited amounts of liver tissues, we need to share these tissues among several projects. **After electrophoresis and before immuno-blotting, filters were cut into several pieces according to the molecular weight of the targeted proteins to be detected. The immunoblot was then preformed with different antibody for each piece.** Therefore, the original raw blots could not be from the top to the bottom of the whole electrophoresis gels.

Figure 5

Twist in Figure 5A (The far right-sided lane was control from cell line; not shown in Figure 5)

N: non-cancerous part; T: cancerous part

Original raw blot:

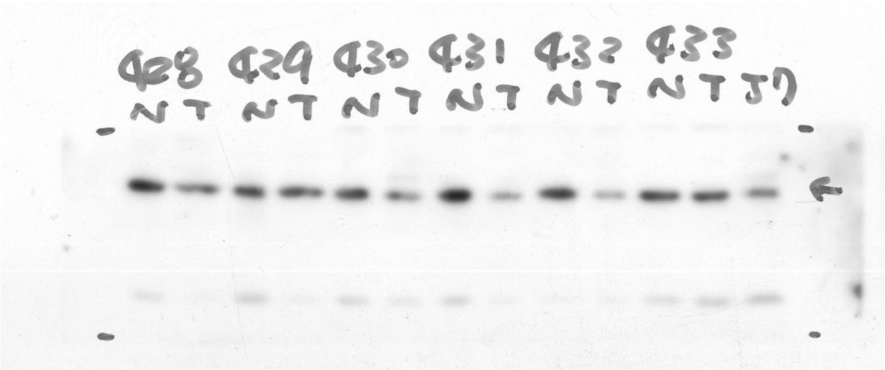

Densitometry quantification

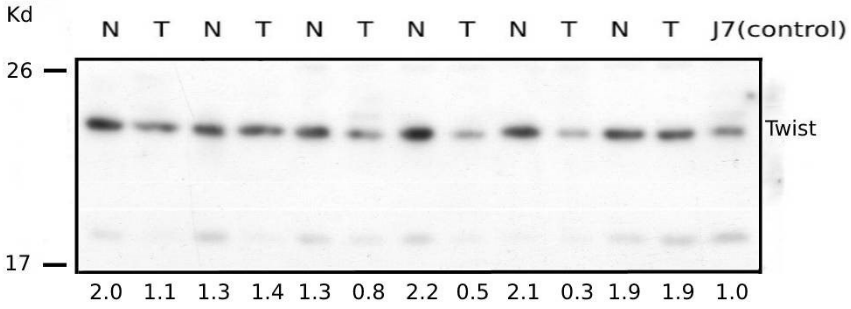

Twist in Figure 5B

Original raw blot

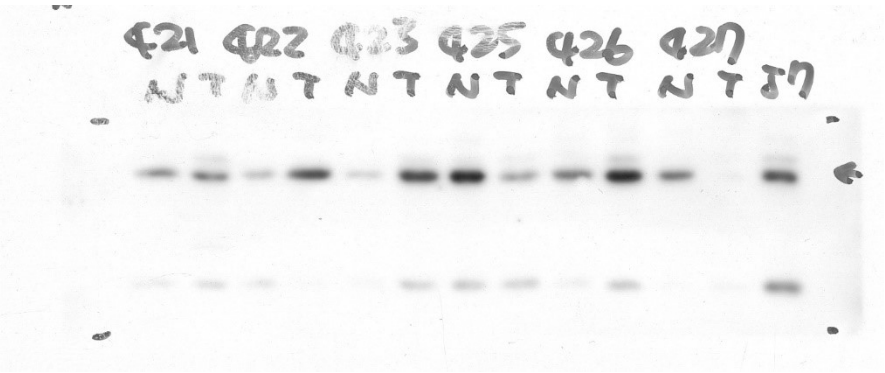

Densitometry quantification

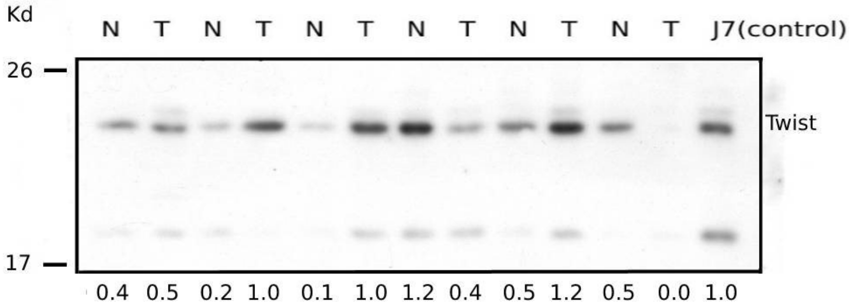

Twist in Figure 5C

Original raw blot

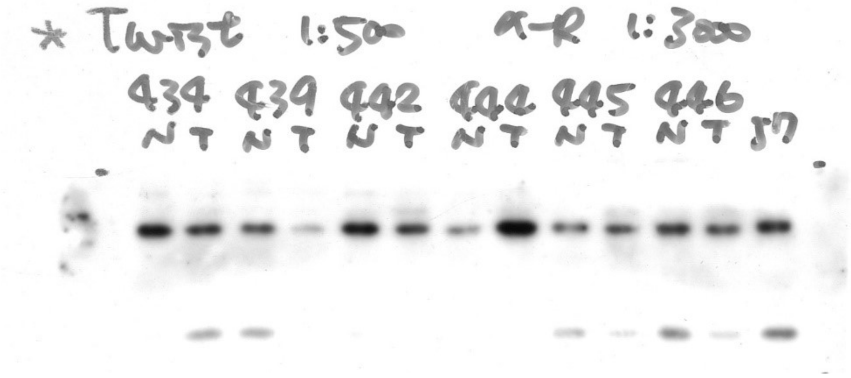

Densitometry quantification

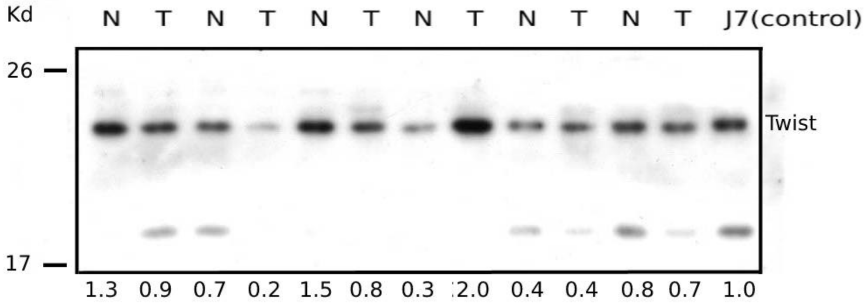

Slug in Figure 5A

Original raw blot

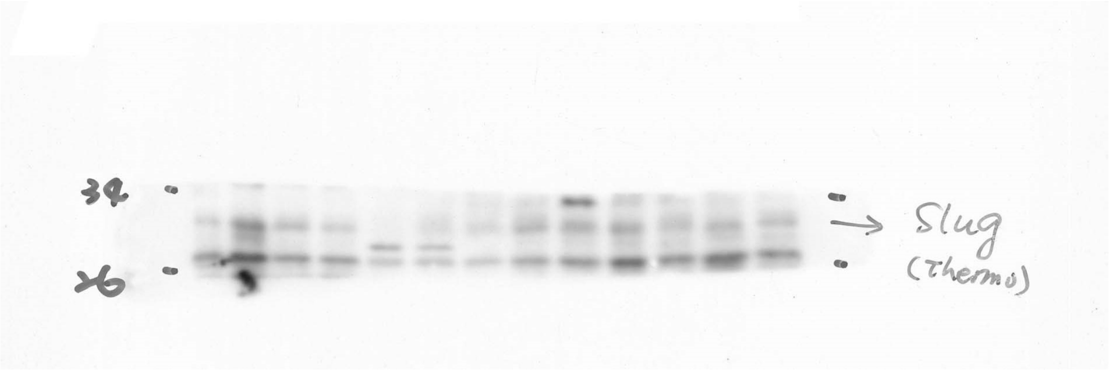

Densitometry quantification

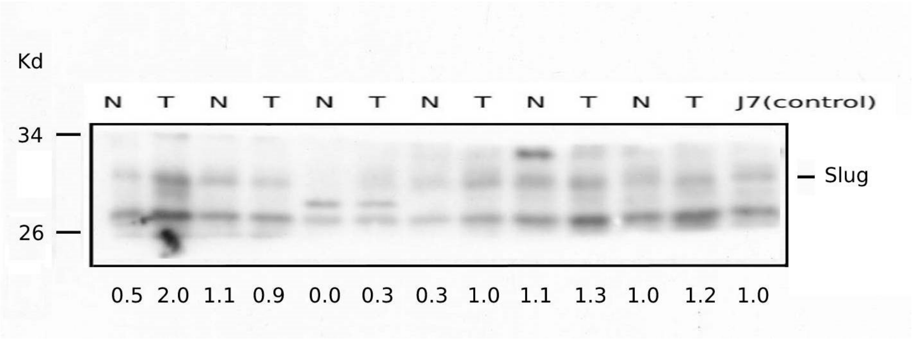

Slug in Figure 5B

Original raw blot

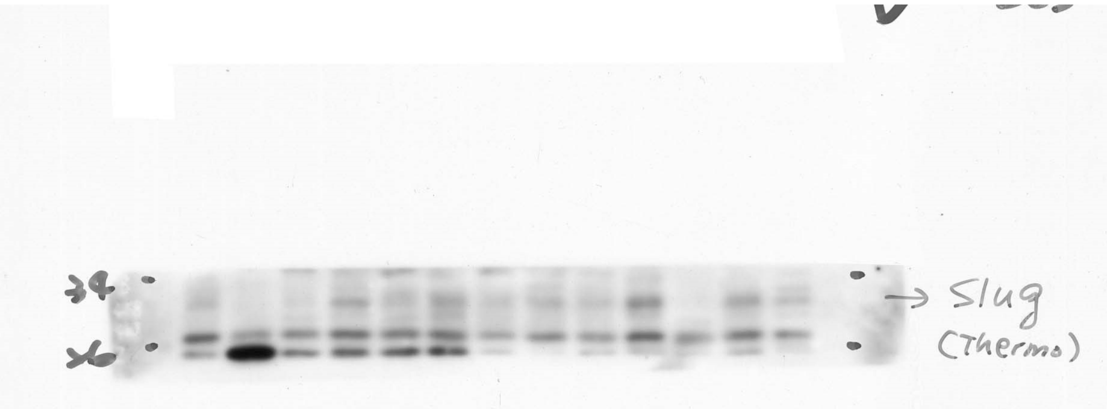

Densitometry quantification

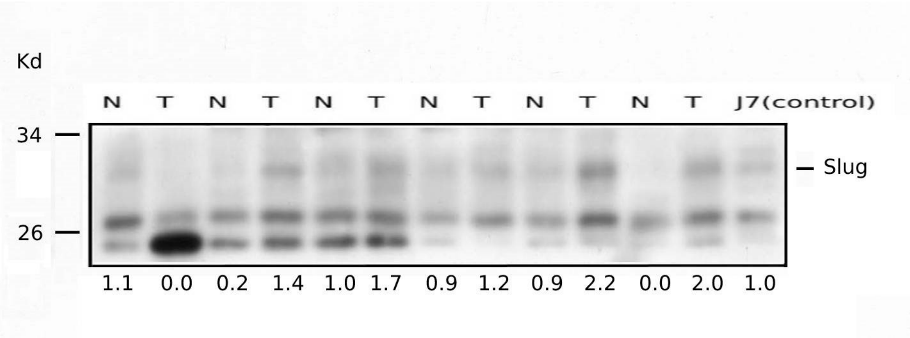

Slug in Figure 5C

Original raw blot

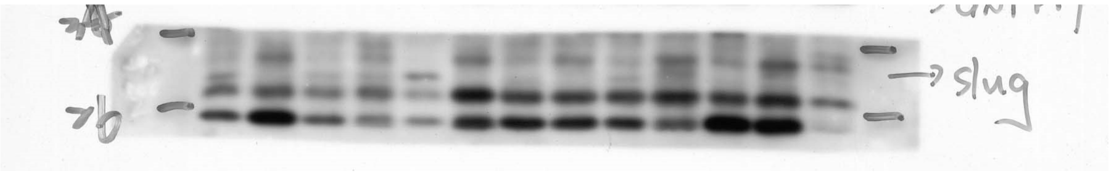

Densitometry quantification

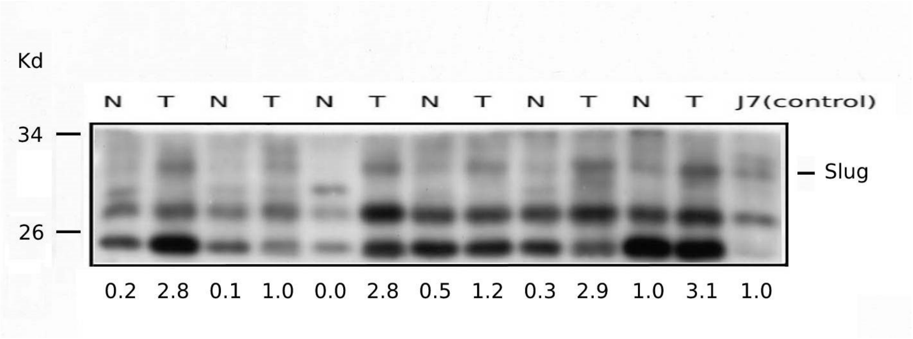

GAPDH in Figure 5A

Original raw blot

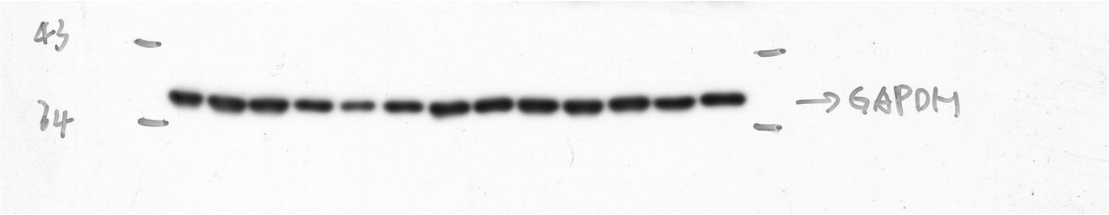

Densitometry quantification

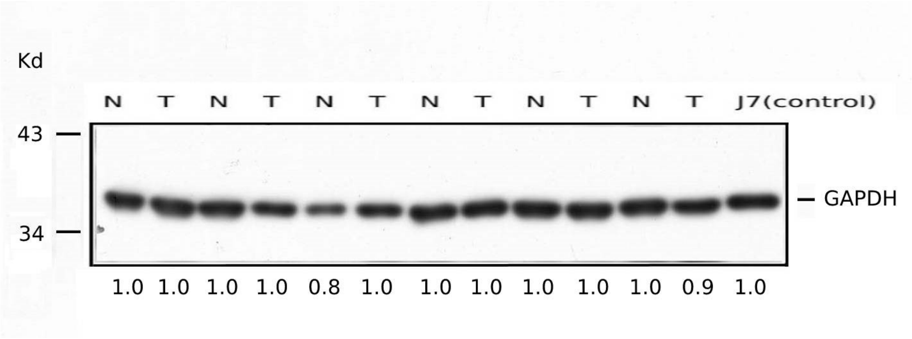

GAPDH in Figure 5B

Original raw blot

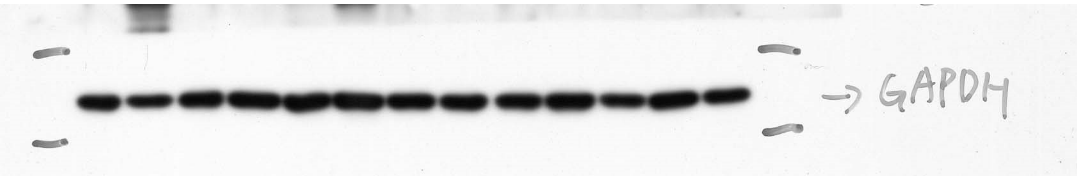

Densitometry quantification

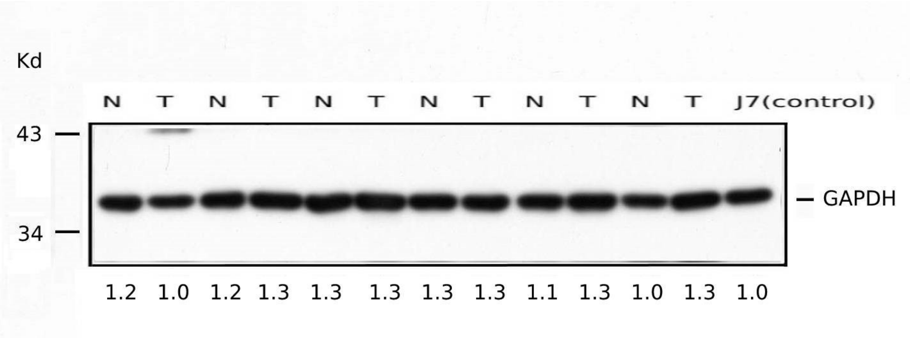

GAPDH in Figure 5C

Original raw blot

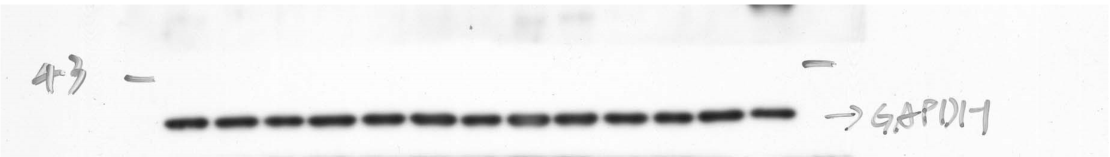

Densitometry quantification

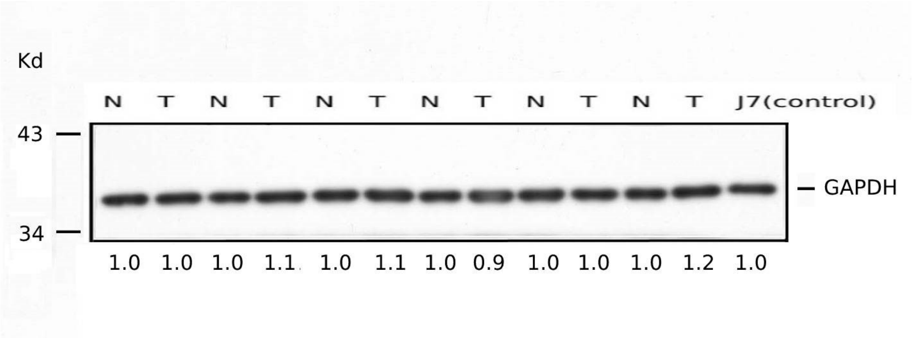

Supplement: Supplementary file 1 [file cancers-13-00538-s001.pdf]
